# Multiplex polymerase chain reaction detection of *Streptococcus pneumoniae and Haemophilus influenzae* and their antibiotic resistance in patients with community-acquired pneumonia from southwest Iran

**DOI:** 10.1186/s12866-021-02408-7

**Published:** 2021-12-14

**Authors:** Ahmad Farajzadeh Sheikh, Robab Rahimi, Hossein Meghdadi, Ameneh Alami, Morteza Saki

**Affiliations:** 1 Department of Microbiology, Faculty of Medicine, Ahvaz Jundishapur University of Medical Sciences, Ahvaz, Iran; 2 Infectious and Tropical Diseases Research Center, Health Research Institute, Ahvaz Jundishapur University of Medical Sciences, Ahvaz, Iran; 3 Student Research Committee, Ahvaz Jundishapur University of Medical Sciences, Ahvaz, Iran

**Keywords:** Community-acquired pneumonia, CAP, *Haemophilus influenzae*, Multiplex polymerase chain reaction, *Streptococcus pneumoniae*

## Abstract

**Background:**

This study aimed to evaluate the occurrence of *Streptococcus pneumoniae* and *Haemophilus influenzae* in sputum of patients with community-acquired pneumonia (CAP) using culture and multiplex polymerase chain reaction (M-PCR) methods and to survey the antibiotic resistance patterns of aforesaid isolates.

**Result:**

In total, 23.9 % (*n* = 22/92) of sputum samples showed positive results in the culture method. *S. pneumoniae* and *H. influenzae* were isolated from 15 (16.3 %) and 7 (7.6%) samples, respectively. Using M-PCR, 44 (47.8 %) samples were positive for *S. pneumoniae* and *H. influenzae*. Of these, *S. pneumoniae* and *H. influenzae* were detected in 33 (35.8%) and 11 (11.9%) of the sputum samples, respectively*.* The sensitivity, specificity, and accuracy rates of PCR in detection of *S. pneumoniae* in comparison with culture method were 100, 76.6, and 83.6%, respectively. While, the sensitivity, specificity, and accuracy rates of PCR in detection of *H. influenzae* in comparison with culture method were 100, 95.3, and 95.8%, respectively. Out of 11 isolates of *H. influenzae*, two strains confirmed as *H. influenzae* type b (Hib) and 3 isolates were type f. However, 6 isolates were non-typable. The co-trimoxazole and amoxicillin/clavulanate were the less effective antibiotics against *S. pneumonia* and *H. influenzae*, respectively. Ceftriaxone with 13.3% resistance rates was the most effective antibiotic against *S. pneumoniae*, while, clarithromycin, ceftriaxone, and gentamicin with resistance rates of 28.6% for each one were the most effective chemicals against *H. influenzae* isolates.

**Conclusion:**

In this study, the prevalence of *S. pneumoniae* was more than *H. influenzae* using culture and M-PCR methods. The M-PCR provided better efficiency in detecting the bacterial agents in CAP patients compared to culture method. This method can improve the early detection of pathogens contributed to CAP. The drug resistant *S. pneumoniae* and *H. influenzae* indicated the need to develop a codified monitoring program to prevent further spread of these strains.

## Background

Over 3 million deaths each year are caused by community-acquired pneumonia (CAP). Also, this infection remains a major challenge to physicians because the highest mortality rates occur in the first days of patient admission [1, 2]. *Streptococcus pneumoniae* and *Haemophilus influenzae* are two main bacteria that contributed to CAP [3]. Although many pathogens can cause CAP, *S. pneumoniae* is the most commonly reported bacterium among adults and young children [4]. This Gram-positive pathogen can colonize the nasopharyngeal region without causing significant clinical indications. In developing and underdeveloped nations, *S. pneumoniae* causes more than 14 million invasive infections and nearly one million mortalities in children each year [5].

*H. influenzae* is the second most frequently reported bacterium in patients with CAP. This Gram-negative bacterium exists in two forms: encapsulated (typeable) and unencapsulated (non-typeable) *H. influenzae* (NTHi) [6, 7]. Encapsulated *H. influenzae* includes 6 serotypes a, b, c, d, e and f [8–10]. *H. influenzae* type b (Hib) is a prevalent cause of serious illness, practically in children under the age of 5 years. Before vaccination, the majority of cases of bacterial meningitis in children under 5 years of age were due to Hib, with more than 83% of cases occurring in children under 2 years of age. While the number of cases of Hib has decreased significantly over the last three decades, its impact is still significant. Approximately 340,000 severe cases of Hib infection were reported globally in 2015 among children aged under 5 years old, with most cases (76%) manifesting as pneumonia and 29,600 deaths linked to the Hib infection [9].

As the CAP infection is life-threatening, rapid laboratory diagnosis and immediate treatment is important to control the disease and save the patient life. The culture method is the gold standard in the detection of bacterial pathogens that cause CAP infection. However, it has some limitations. First, it is time-consuming (24-72 h) and the second is low bacterial growth in people who have received antibiotics [11, 12]. Hence, using molecular techniques such as polymerase chain reaction (PCR) that are rapid and sensitive, can improve the speed and accuracy of detection of CAP pathogens [13].

Although the antibiotic resistance has increased in most parts of the world and among different bacteria, the true extent of antibiotic non-susceptibility among respiratory pathogens in southwestern Iran has not been well studied. Recent studies from China [14] and Taiwan [15] have reported the emergence of multidrug-resistant *S. pneumoniae* and *H. influenzae* strains.

This study aimed to evaluate the occurrence of *S. pneumoniae* and *H. influenzae* and their antibiotic resistance patterns in sputum samples of patients with CAP from southwest Iran.

## Materials and methods

### Ethics statement

This study was approved by the Research Ethics Committee (IR.AJUMS.REC.1394.458) of the Ahvaz Jundishapur University of Medical Sciences, Ahvaz, Iran following the Declaration of Helsinki. Written informed consent was obtained from each patient before collecting sputum samples.

### Sample collection

This cross-sectional study was conducted during the two years 2014 to 2016 at the teaching hospitals of Ahvaz city, southwest Iran. The diagnosis of CAP patients was confirmed by specialists in respiratory and lung infectious diseases of the hospitals. Ninety-two sputum samples were collected from patients who were selected based on their clinical symptoms, chest x-ray, and laboratory tests including positive C-reactive protein (CRP), elevated procalcitonin, and leukocytosis. Inclusion criteria were the presence of pneumonia on the basis of a clinical assessment including fever, cough, sputum production, pleuritic chest pain, and dyspnea. Patients who got antibiotic therapy within the last three days were excluded from this study.

### Culture and microbiologic testing

The sputum samples were taken in a sterile container and were transported to the laboratory within less than one hour and immediately analyzed microscopically by Gram staining. The samples that had more than 25 polymorphonuclear cells and less than 10 epithelial cells per low power field were included in this study as suitable sputum samples. After Gram staining and direct microscopic examination, sputum samples were cultured on sheep blood agar and chocolate agar (Merck, Germany) and incubated at 35 °C with 5 to 10 % CO_2_. After 48 hours of incubation, the suspected colonies of *S. pneumoniae* and *H. influenzae* were identified by a panel of standard biochemical and bacteriological tests. For *S. pneumonia*, Gram staining, colony morphology, catalase, hemolysis on blood agar plate, bile solubility, and optochin tests were included. For *H. influenzae*, Gram staining, colony morphology, growth on chocolate agar, oxidase, X factor (hemin) and V factor (nicotinamide-adenine-dinucleotide, NAD) requirement were included [16].

### Antibiotic susceptibility test

Antimicrobial susceptibility testing was carried out using disc diffusion method according to the Clinical and Laboratory Standards Institute (CLSI) guidelines [17]. The following antibiotics were used for *S. pneumoniae*: ceftriaxone (CRO, 30 μg), erythromycin (ERY, 15 μg), co-trimoxazole (SXT, 1.25/23.75 μg), clarithromycin (CLR, 15 μg), and amoxicillin/clavulanate (AMC, 20/10 μg). Also, the following antibiotics were used for *H. influenzae*: ceftriaxone (CRO, 30 μg), co-trimoxazole (SXT, 1.25/23.75 μg), clarithromycin (CLR, 15 μg), and amoxicillin/clavulanate (AMC, 20/10 μg). Mueller-Hinton agar (Merck, Germany) with 5% sheep blood and *Haemophilus* test medium (HTM) (Condalab, Spain) were used for *S. pneumonia* and *H. influenzae*, respectively.

### Multiplex polymerase chain reaction (M-PCR) technique

DNA extraction was performed from sputum samples by High Pure PCR Template Preparation Kit (Roche Diagnosis, Mannheim, Germany) according to manufacturers, instructions. The primers used for M-PCR were specific for *lytA* gene (395bp) of *S. pneumoniae* and P6 protein (273bp) of *H. influenzae* (Table 1) [18, 19]*.* The PCR reaction composed of 0.5 μl of each primer, 12.5 μl of Mastermix [50 mM KCl, 10 mmol Tris-HCl (pH 8.3), 0.2 mM each dNTP, 1.5 mM MgCl_2_, 1U Taq DNA polymerase] (Genet Bio, Korea), 5 μl of template DNA, and DNA/RNA free water to reach the final volume of 25 μl. The programs of PCR were as follows: 5 minutes at 95 °C followed by 35 cycles (denaturation at 95 °C for 30 seconds, annealing at 54 °C for 45 seconds and extension at 72 °C for 40 seconds), and the final extension at 72 °C for 7 minutes. *S. pneumoniae* (ATCC® 33400™) and *H. influenzae* (ATCC® 33391™) standard strains were used as positive controls. Also, the *E. coli* ATCC® 11775™ and master mix without DNA template were used as two negative controls in each PCR run. The electrophoresis (100 V, 40 min) was performed using a 1.5 % agarose gel for the detection of amplified products.

**Table 1:** The primer sets used in this study

Target	Primer sequence	Amplicon size	Reference
*lytA*	5´-GGCTACTGGTACGTACATTC-3´ 5´-AATCAAGCCATCTGGCTCTA-3´	395bp	[18]
P6	5´- ACTTTTGGCGGTTACTCTGT-3´ 5´- TGTGCCTAATTTACCAGCAT-3´	273bp	[19]
*bexA*	HI-1-5'-CGTTTGTATGATGTTGATCCAGAC-3' HI-2-5'-TGTCCATGTCTTCAAAATGATG-3'	343bp	[19]
Type a	a1-5'-CTACTCATTGCAGCATTTGC-3' a2-5'-GAATATGACCTGATCTTCTG-3'	250bp	[20]
Type b	b1-5'-GCGAAAGTGAACTCTTATCTCTC-3' b2-5'-GCTTACGCTTCTATCTCGGTGAA-3'	480bp	[20]
Type c	c1-5'-TCTGTGTAGATGATGGTTCA-3' c2-5'-CAGAGGCAAGCTATTAGTGA-3'	250bp	[20]
Type d	d1-5'-TGATGACCGATACAACCTGT-3' d2-5'-TCCACTCTTCAAACCATTCT-3'	150bp	[20]
Type e	e1-5'-GGTAACGAATGTAGTGGTAG-3' e2-5'-GCTTTACTGTATAAGTCTAG-3'	1350bp	[20]
Type f	f1-5'-GCTACTATCAAGTCCAAATC-3' f2-5'-CGCAATTATGGAAGAAAGCT-3'	450bp	[20]

### Capsular typing of *H. influenzae* by PCR

To differentiate encapsulated *H. influenzae* strains from NTHi strains, the *bexA* gene was amplified. Capsular typing performed using six specific primer sets which are listed in Table 1 [19, 20]. The following standard strains were used for positive control: *H. influenzae* type a (ATCC® 9006™), *H. influenzae* type b (ATCC® 9795™), *H. influenzae* type c (ATCC® 9007™), *H. influenzae* type d (ATCC® 9332™), *H. influenzae* type e (ATCC® 8142™), and *H. influenzae* type f (ATCC® 9833™). *H. influenzae* isolates containing the *bexA* gene were considered as encapsulated *H. influenzae* and were further classified based on the presence of each of the capsular genes*.* The NTHi strains were those that lacking both *bexA* gene and any of the capsular genes. The PCR reactions composed of 0.5 μl of each primer, 12.5 μl of Mastermix [50 mM KCl, 10 mmol Tris-HCl (pH 8.3), 0.2 mM each dNTP, 1.5 mM MgCl2, 1U Taq DNA polymerase] (Genet Bio, Korea), 5 μl of template DNA, and DNA/RNA free water to reach the final volume of 25 μl. PCR conditions were as follows: an initial denaturation at 95 °C for 5 min, followed by 35 cycles of denaturation at 95 °C for 30 s, annealing (Table 2) for 30 s, extension at 72 °C for 30 s, and final extension at 72 °C for 5 min. The products of each PCR assay were analyzed by gel electrophoresis on 1.5% agarose.

**Table 2:** Test performance of multiplex polymerase chain reaction (M-PCR) in detection of *Streptococcus pneumoniae* and *Haemophilus influenzae* compared to culture method

	Culture for *Streptococcus pneumoniae*			Culture for *Haemophilus influenzae*		
	Positive	Negative	Total	Positive	Negative	Total
**PCR Positive**	15 (16.3%)	18 (19.5%)	33 (35.8%)	7 (7.6%)	4 (4.3%)	11 (11.9%)
**PCR Negative**	0 (0.0%)	59 (64.2%)	59 (64.2%)	0 (0.0%)	81 (88.1%)	81 (88.1%)
**Total**	15 (16.3%)	77 (83.7%)	92 (100%)	7 (7.6%)	85 (92.4%)	92 (100%)
**Sensitivity (%)** **(95% CI)**	100.0% (89.4 to 100.0%)			100.0% (71.5 to 100.0%)		
**Specificity (%)** **(95% CI)**	76.6% (65.6 to 85.5%)			95.3% (88.4 to 98.7%)		
**Positive predictive value (%)** **(95% CI)**	64.7% (55.0 to 73.3%)			73.3% (51.4 to 87.7%)		
**Negative predictive value (%)** **(95% CI)**	100.0%			100.0%		
**Positive likelihood ratio (%)** **(95% CI)**	4.3% (2.9 to 6.4%)			21.3% (8.2 to 55.3%)		
**Negative likelihood ratio (%)** **(95% CI)**	0.0%			0.0%		
**Test accuracy (%)** **(95% CI)**	83.6% (75.4 to 90.0%)			95.8% (89.7 to 98.9%)		

### Statistical analysis

The descriptive statistic tests were performed in SPSS version 21.0 (Armonk, NY, USA), and a level of significance of *P-*value < 0.05 was used. Continuous data was compared using 95 % confidence intervals (CI).

## Results

### Prevalence of *S. pneumoniae* and *H. influenzae* isolates by culture and M-PCR

A total of 92 sputum samples were collected from 50 (54.3%) males and 42 (45.7%) females suffered from CAP. The patients’ ages ranged from 25 to 93 with the mean age of 59 years. Out of 92 sputum samples, 22 (23.9%) were culture positive, from which 15 (16.3%) and 7 (7.6%) samples were positive for *S. pneumoniae* and *H. influenzae*, respectively. Among the 92 samples, 44 (47.8%) were positive in M-PCR analysis, from which 33 (35.8%), 11 (11.9%), and 3 (3.2%) samples were positive for S*. pneumoniae*, *H. influenzae*, and both pathogens, respectively*.* The detection rates of both pathogens were much higher in M-PCR method compared to the culture method (*P* < 0.005) (Table 2). The sensitivity, specificity, and accuracy rates of M-PCR in detection of *S. pneumoniae* in comparison with culture method were 100, 76.6, and 83.6%, respectively. While, the sensitivity, specificity, and accuracy rates of PCR in detection of *H. influenzae* in comparison with culture method were 100, 95.3, and 95.8%, respectively.

### Capsular typing of *H. influenzae*

All 11 positive samples of *H. influenzae* in M-PCR were evaluated by another run of PCR amplification for the *bexA* and capsular genes (*a*, *b*, *c*, *d*, *e* and *f*). Out of 11 isolates of *H. influenzae,* two strains containing both the *bexA* gene and capsular gene b and confirmed as Hib strains. Three isolates showed the *bexA* and capsular gene f amplification. However, 6 isolates lacking both *bexA* and any of the capsular genes and were considered as NTHi.

### Antimicrobial susceptibility patterns

The antibiotic susceptibility patterns of the S*. pneumonia* and *H. influenzae* isolates are shown in Table 3. Ceftriaxone and co-trimoxazole with 13.3 and 73.3% of resistance rates were the most and the less effective antibiotics against *S. pneumoniae*, respectively. Clarithromycin, ceftriaxone, and gentamicin with resistance rates of 28.6% for each one were the most effective chemicals against *H. influenzae* isolates. While, the amoxicillin/clavulanate (resistance rate 85.7%) was the weakest drug.

**Table 3:** Sensitivity patterns of *Streptococcus pneumoniae* and Haemophilus influenza isolates

Antimicrobial agents	Susceptible/Resistant	***S. pneumoniae*** (***n***=15)	***H. influenzae*** (***n***=7)
Ciprofloxacin	S	Not recommended by CLSI	3 (42.9%)
	R		4 (57.1%)
Amoxicillin/clavulanate	S	10 (66.7%)	1 (14.3%)
	R	5 (33.3%)	6 (85.7%)
Clarithromycin	S	6 (40%)	5 (71.4%)
	R	9 (60%)	2 (28.6%)
Ceftriaxone	S	13 (86.7%)	5 (71.4%)
	R	2 (13.3%)	2 (28.6%)
Co- trimoxazole	S	4 (26.7%)	4 (57.1%)
	R	11 (73.3%)	3 (42.9%)
Erythromycin	S	7 (46.7%)	Not recommended by CLSI
	R	8 (53.3%)	

*CLSI* Clinical and laboratory standards institute

## Discussion

Conventional methods, such as culture and serology are not always adequate to detect CAP pathogens. Rapid diagnosis of etiologic agents of CAP based on phenotypic methods is difficult; therefore, new diagnosis methods are needed [21]. It is well known that sensitivity and specificity of routine culture is low for identification of CAP pathogens. Thus, more sensitive and rapid diagnostic methods, such as molecular method, could possibly be useful for detect of CAP pathogens [22].

In this study, both culture and M-PCR methods were used for detection of two bacterial causes (*S. pneumoniae* and *H. influenzae*) of CAP. The results showed that the detection rate of M-PCR method (47.8%) was higher than culture method [23.9%]. In line with these findings, Maleki et al. [23] from Iran showed the superiority of molecular techniques such as real-time PCR than culture in detection of *S. pneumoniae* and *H. influenzae* from oropharynx and nasal cavity. Also, in another study by et al. [24] from United Kingdom, the detection rates of molecular method were 9.4–26.2% higher than culture for *S. pneumoniae*, *Moraxella catarrhalis*, and *H. influenzae* that was in agreement with this study. Also, in this study the accuracy rate of M-PCR in detection of *H. influenzae* was higher than *S. pneumoniae* that was in line with the previous study by Bjarnason et al. [25] from Iceland.

This study revealed a 100% sensitivity of M-PCR in detection of *H. influenzae* and *S. pneumoniae* compared to culture method. In contrast to the current research, Gillis et al. [26] showed that PCR based method had poor sensitivity in detection of *S. pneumoniae* in nasopharyngeal swabs of CAP patients. This discrepancy may be due to the differences in sample types, as we used sputum samples in the current study. The M-PCR method had lower specificity in detection of *S. pneumoniae* than *H. influenzae* in this study. Due to the existence of autolysin gene (*lytA*) in other oral flora streptococci, this cross-reactivity may be reduce the M-PCR specificity for detection of *S. pneumoniae* in sputum samples [25]. Shakib et al. [27] from Iran showed the 100% sensitivity of real-time PCR for detection of *S. pneumoniae* in sputum samples using the *lytA* gene that was similar to our results. In another study by Fan et al. [28] from China, a one-step M-PCR assay detecting the *ompP6* and the *bexA* genes of *H. influenzae* was compared with culture and serum agglutination test. The results of M-PCR showed the sensitivity and specificity of 100 and 99.8%, respectively. This findings were close to our results that exhibited a sensitivity of 100% and the specificity of 95.3% of M-PCR in detection of *H. influenzae*.

In this study the culture method revealed prevalence rates of 16.3 and 7.6% for *S. pneumoniae* and *H. influenzae*, respectively. Using culture method, prevalence rates of 6.5% from Iran [23], 16% from Iceland [25], and 18.4% from China [28] has been reported in previous reports for *H. influenzae*. Also, occurrence rates of 11.4% from Iran [23] and 20% from Iceland [25] has been reported for *S. pneumoniae* in previous researches. According to the study of Aydemiret et al. [29] from Turkey, the isolation rates of *S. pneumoniae* and *H. influenzae* from sputum, nasopharyngeal swabs and bronchoalveolar lavage (BAL) fluid samples by culture method were 16.2 and 0.0%, respectively. This frequency rate of *S. pneumonia* was similar to the current study. In contrast to the current study, Costa et al. [6] from Portugal, reported a higher frequency rates of *H. influenzae* (21.4%) than *S. pneumoniae* (14.1%) in sputum/BAL samples using culture method.

In the current research, using M-PCR method, the incidence rates of 35.8, 11.9, and 3.2% were revealed for *S. pneumoniae*, *H. influenzae*, and both pathogens, respectively. Fan et al. [28] from China reported a higher prevalence (19.3%) for *H. influenzae* in nasopharyngeal secretion of Children suffered from respiratory infections. Aydemir et al. [29] from Turkey, reported frequency rates of 15.2, 12.7, and 14.7% for *S. pneumoniae*, *H. influenzae*, and both pathogens coinfection, respectively. In a study from United Kingdom [30], more *H. influenzae* (40.2%) was detected than *S. pneumoniae* (35.6%) in patients with CAP that was in contrast to the current results. So far, scarce studies have examined the prevalence of CAP-related pathogens in Iran, most of which focus on only a small number of bacterial or viral pathogens. In a study by Naderi et al. [31] from Mashhad, Iran, the *S. pneumoniae* (24.4%) was the most prevalent pathogen in CAP patients using conventional culture method and standard BACTEC device. However, they reported a lower prevalence rate for *S. pneumoniae* than our study. Also, inconsistent to the current research, Temesgen et al. [32] from Ethiopia, reported higher prevalence rates for *S. pneumoniae* (35.9%) and *H. influenzae* (8.4%) using conventional culture and biochemical tests. These variations among several studies can be due to differences in geographical area, type of detection method, sample size, type of studied sample, and age range of the study population.

Another finding of the current study was the prevalence rates of 2.1 and 6.5% for Hib and NTHi using PCR method, respectively. Also, 3.2% of isolates were *H. influenzae* type f. Our results were consistent with previous evidence that reported a 2 to 12% prevalence of CAP due to NTHi [10]. Hib is a leading cause of serious illness in children under the age of 5. Previous report from Iran showed the prevalence rate of 8% for Hib in clinical samples [20]. The present study is one of a handful of studies that examined the prevalence of different capsular types of *H. influenzae* in Iran. In this study, a, c, d, and e types were not observed. In another study from Iran, the b, e, and f capsular types were reported in *H. influenzae* isolates [33]. There has been evidence from both developed and developing countries that introducing the Hib vaccination has reduced its carrier numbers. In November 2014, the pentavalent vaccine which contains diphtheria, tetanus, whole cell pertussis, hepatitis B, and *H. influenzae* type b entered the Iranian National Immunization Plan. Vaccination against Hib may reduce Hib colonization, while increasing other serotypes [8, 34] The technique used to identify the *H. influenzae* capsular type may affect the results. Because in some studies, slide agglutination serotyping-based methods and in some others the molecular methods are used [33].

One of the strength of the current study was the determination of antibiotic resistance patterns of *S. pneumoniae* and *H. influenzae* isolates against some routine antibiotics. *S. pneumoniae* showed most susceptibility against ceftriaxone (86.7%) and amoxicillin/clavulanate (66.7%) which was in agreement with previous studies by Temesgen et al. [32] and Akter et al. [35] who showed the good efficacy of two aforesaid antibiotics against *S. pneumoniae*. However, the co-trimoxazole with susceptibility rate of 26.7% was the less effective antibiotic against *S. pneumoniae*. Conversely, Temesgen et al. [32] reported an 8.3% of resistance rate among *S. pneumoniae* isolates toward the co-trimoxazole. In another study from Iran, the susceptibility rates of *S. pneumoniae* isolates in patients with non-meningitis invasive disease were 90.4 and 33.3% for ceftriaxone and co-trimoxazole, respectively [36]. Also, the susceptibility rates of *S. pneumoniae* isolates for erythromycin and clarithromycin were 46.7 and 40%, respectively. The sensitivity rate for erythromycin was higher than a previous report by Houri et al. [36] from Iran who stated the rate of 23.8%, but lower than a report by Temesgen et al. [32] who showed susceptibility rate of 96.7%. However, in a study by Zhao et al. [37] from China, 95.2 and 92.5% of *S. pneumoniae* isolates were resistant to erythromycin and clarithromycin, respectively. *S. pneumoniae* is mainly treated with beta-lactams and macrolides, while fluoroquinolones occupied the third treatment choice. There is a major concern in the world regarding beta-lactams and macrolides resistant *S. pneumoniae* isolates. *S. pneumoniae* was recently ranked among the 12 bacteria for which new treatments are severely needed by the World Health Organization [38].

The current research showed the good efficacy of clarithromycin and ceftriaxone (more than 70%) against *H. influenzae* isolates. While, the amoxicillin-clavulanate (resistance rate 85.7%) was the weakest treatment choice. In a previous study by Shooraj et al. [39] from Iran the susceptibility rate of 80% was reported for ceftriaxone that was almost close to this research. Also, our isolates showed the resistance rates of 57.1, and 42.9% for ciprofloxacin and co-trimoxazole, respectively. In contrast to the current study, Shooraj et al. [39] stated the quinolones as the most effective antibiotics against *H. influenza*e. Also, they showed a higher resistance rate (57.7%) for co-trimoxazole compared to our findings. In another study by Boroumand et al. [40] from Iran, all *H. influenza* isolates were sensitive to co-trimoxazole. Also, they reported resistance rates of 50 and 45% for ceftriaxone and ciprofloxacin, respectively. In a recent meta-analysis from Iran, the resistance rates of *H. influenzae* to various antibiotics were as follows: co-trimoxazole 53%, erythromycin 40.3%, ciprofloxacin 30.8%, ceftriaxone 33.1%, and amoxicillin-clavulanate 11.8%. As it is obvious, the rate of amoxicillin-clavulanate resistance in the current study was much higher than the Iranian average. Also, our results were in contrast to previous reported resistance rate for amoxicillin-clavulanate (0.0%) from Ethiopia [32]. Also, in the later study [32], a lower and an equal resistance rates were reported against ciprofloxacin (35.7%) and ceftriaxone (28.6%) against *H. influenzae* isolates compared to the current research.

### Limitations

It is important to acknowledge some limitations of this study. This research could not include all clinical and high risk parameters of patients that may affect the etiology of CAP infection. Also, the focus of this study was based on the prevalence of *S. pneumoniae* and *H. influenzae*. Hence, the other bacterial or viral pathogens were not investigated in CAP patients. Also, this study was limited to CAP patients who produced sputum and no blood cultures and urinary antigen test were included. Sputum is still an effective specimen type when investigating CAP microbiologically, despite its shortcomings.

## Conclusions

In this study, the M-PCR showed good sensitivity and specificity compared to culture method in detection of *H. influenzae* and *S. pneumoniae* in sputum samples of CAP patients. The M-PCR provided better efficiency in detecting the bacterial agents in CAP patients compared to culture method. It is recommended that this technique be used in addition to the culture method in the detection of pathogens involved in the CAP infection. Also, the prevalence of *S. pneumoniae* was higher than *H. influenzae* in CAP patients. The Hib was detected in 2.1% of patients. The *H. influenzae* and *S. pneumoniae* isolates showed various resistance rates against studied antibiotics. The drug resistant *S. pneumoniae* and *H. influenzae* indicated the need to develop a codified monitoring program to prevent further spread of these strains.

## Data Availability

All data generated or analyzed during this study are included here and are available from the corresponding author on reasonable request.

## Abbreviations

CAP: Community-acquired pneumonia
CLSI: Clinical Laboratory Standard Institute
Hib: *Haemophilus influenzae* type b
M-PCR: Multiplex polymerase chain reaction
NTHi: Non-typeable *Haemophilus influenzae*
