# Correction to: Multiplex polymerase chain reaction detection of *Streptococcus pneumoniae and Haemophilus influenzae* and their antibiotic resistance in patients with community-acquired pneumonia from southwest Iran

**DOI:** 10.1186/s12866-022-02449-6

**Published:** 2022-02-18

**Authors:** Ahmad Farajzadeh Sheikh, Robab Rahimi, Hossein Meghdadi, Ameneh Alami, Morteza Saki

**Affiliations:** 1 Department of Microbiology, Faculty of Medicine, Ahvaz Jundishapur University of Medical Sciences, Ahvaz, Iran; 2 Infectious and Tropical Diseases Research Center, Health Research Institute, Ahvaz Jundishapur University of Medical Sciences, Ahvaz, Iran; 3 Student Research Committee, Ahvaz Jundishapur University of Medical Sciences, Ahvaz, Iran

**Correction to: BMC Microbiol 21, 343 (2021)**

**https://doi.org/10.1186/s12866-021-02408-7**

Following the publication of the original article [1], author Hossein Meghdadi was not captured as corresponding author. On this paper, he is considered as the 2^nd^ corresponding author, as shown in the author group section above.

The original article has been corrected.
